# Gallbladder Cancer Risk and Indigenous South American Mapuche Ancestry: Instrumental Variable Analysis Using Ancestry-Informative Markers

**DOI:** 10.3390/cancers15164033

**Published:** 2023-08-09

**Authors:** Linda Zollner, Felix Boekstegers, Carol Barahona Ponce, Dominique Scherer, Katherine Marcelain, Valentina Gárate-Calderón, Melanie Waldenberger, Erik Morales, Armando Rojas, César Munoz, Javier Retamales, Gonzalo De Toro, Allan Vera Kortmann, Olga Barajas, María Teresa Rivera, Analía Cortés, Denisse Loader, Javiera Saavedra, Lorena Gutiérrez, Alejandro Ortega, Maria Enriqueta Bertrán, Leonardo Bartolotti, Fernando Gabler, Mónica Campos, Juan Alvarado, Fabricio Moisán, Loreto Spencer, Bruno Nervi, Daniel Carvajal, Héctor Losada, Mauricio Almau, Plinio Fernández, Jordi Olloquequi, Alice R. Carter, Juan Francisco Miquel Poblete, Bernabe Ignacio Bustos, Macarena Fuentes Guajardo, Rolando Gonzalez-Jose, Maria Cátira Bortolini, Victor Acuña-Alonzo, Carla Gallo, Andres Ruiz Linares, Francisco Rothhammer, Justo Lorenzo Bermejo

**Affiliations:** 1 Statistical Genetics Research Group, Institute of Medical Biometry, Heidelberg University, 69120 Heidelberg, Germany; zollner@imbi.uni-heidelberg.de (L.Z.); boekstegers@imbi.uni-heidelberg.de (F.B.); barahona@imbi.uni-heidelberg.de (C.B.P.); scherer@imbi.uni-heidelberg.de (D.S.); garate@imbi.uni-heidelberg.de (V.G.-C.); 2 Division of Proteomics of Stem Cells and Cancer, German Cancer Research Center, 69120 Heidelberg, Germany; 3 Department of Basic and Clinical Oncology, Medical Faculty, University of Chile, Santiago 8380000, Chile; kmarcelain@uchile.cl (K.M.); obarajas@hcuch.cl (O.B.); 4 Research Unit Molecular Epidemiology, Institute of Epidemiology, Helmholtz Zentrum München, German Research Center for Environmental Health, 85764 Neuherberg, Germany; waldenberger@helmholtz-muenchen.de; 5 Hospital Regional de Talca, Talca 3460000, Chile; emoralesm@hospitaldetalca.cl (E.M.); cmunozc@hospitaldetalca.cl (C.M.); 6 Facultad de Medicina, Universidad Católica del Maule, Talca 3460000, Chile; arojasr@ucm.cl; 7 Instituto Nacional del Cáncer, Santiago 7500650, Chile; jretamales@gocchi.org; 8 Hospital de Puerto Montt, Puerto Montt 5480000, Chile; gonzalo.detoro@uach.cl (G.D.T.); allan.vera@araucanianorte.cl (A.V.K.); 9 Escuela de Tecnología Médica, Universidad Austral de Chile sede Puerto Montt, Puerto Montt 5480000, Chile; 10 Hospital Clínico Universidad de Chile, Santiago 8380456, Chile; 11 Hospital del Salvador, Santiago 7500922, Chile; mrivera@hsalvador.cl (M.T.R.); acortes@hsalvador.cl (A.C.); 12 Hospital Padre Hurtado, Santiago 8880456, Chile; dloader@hurtadohosp.cl (D.L.); javiera.saavedra@hurtadohosp.cl (J.S.); 13 Hospital San Juan de Dios, Santiago 8320000, Chile; lorenaf.gutierrez@redsalud.gob.cl; 14 Hospital Regional, Arica 1000000, Chile; alejandro.ortega@hjnc.cl; 15 Unidad Registro Hospitalario de Cáncer, Hospital Base de Valdivia, Valdivia 5090146, Chile; enriqueta.bertran@redsalud.gov.cl; 16 Hospital Base de Valdivia, Valdivia 5090000, Chile; leonardo.bartolotti@redsalud.gob.cl; 17 Hospital San Borja Arriarán, Santiago 8320000, Chile; gablerf@gmail.com (F.G.); monicam.camposm@redsalud.gob.cl (M.C.); 18 Hospital Regional Guillermo Grant Benavente, Concepción 4070386, Chile; jualvarado@udec.cl (J.A.); fabriziomoisan@udec.cl (F.M.); mspencer@udec.cl (L.S.); 19 Departamento de Hematología y Oncología, Escuela de Medicina, Pontificia Universidad Católica de Chile, Santiago 8330077, Chile; bnervi@uc.cl; 20 Facultad de Medicina, Clínica Alemana Universidad del Desarrollo, Santiago 7650568, Chile; dcarvajal@alemana.cl; 21 Departamento de Cirugía, Universidad de la Frontera, Temuco 4780000, Chile; hector.losada@ufrontera.cl; 22 Hospital de Rancagua, Rancagua 2820000, Chile; halmau@clinicaisamedica.cl (M.A.); pfernandez@clinicaisamedica.cl (P.F.); 23 Department of Biochemistry and Physiology, Faculty of Pharmacy and Food Sciences, University of Barcelona, 08028 Barcelona, Spain; jordiolloquequi@ub.edu; 24 Facultad de Ciencias de la Salud, Universidad Autónoma de Chile, Talca 3460000, Chile; 25 MRC Integrative Epidemiology Unit, Population Health Sciences, Bristol Medical School, University of Bristol, Bristol BS8 1UD, UK; alice.carter@bristol.ac.uk; 26 Departamento de Gastroenterología, Escuela de Medicina, Pontificia Universidad Católica de Chile, Santiago 8320000, Chile; jfmiquel@med.puc.cl; 27 Ken and Ruth Davee Department of Neurology and Simpson Querrey Center for Neurogenetics, Northwestern University, Feinberg School of Medicine, Chicago, IL 60611, USA; bernabe.bustos@northwestern.edu; 28 Departamento de Tecnología Médica, Facultad de Ciencias de la Salud, Tarapacá University, Arica 1000815, Chile; mafuentesg@academicos.uta.cl; 29 Instituto Patagónico de Ciencias Sociales y Humanas, Centro Nacional Patagónico, CONICET, Puerto Madryn U9120ACD, Argentina; rolando@cenpat-conicet.gob.ar; 30 Instituto de Biociências, Universidad Federal do Rio Grande do Sul, Puerto Alegre 15053, Brazil; maria.bortolini@ufrgs.br; 31 National Institute of Anthropology and History, Mexico City 06600, Mexico; victor_acuna@inah.gob.mx; 32 Laboratorios de Investigación y Desarrollo, Facultad de Ciencias y Filosofía, Universidad Peruana Cayetano Heredia, Lima 15102, Peru; carla.gallo@upch.pe; 33 Ministry of Education Key Laboratory of Contemporary Anthropology, Collaborative Innovation Center of Genetics and Development, School of Life Sciences and Human Phenome Institute, Fudan University, Shanghai 200434, China; a.ruizlin@ucl.ac.uk; 34 ADES (Anthropologie Bio-Culturelle, Droit, Éthique et Santé), UFR de Médecine, Aix-Marseille University, 13007 Marseille, France; 35 Department of Genetics, Evolution and Environment and UCL Genetics Institute, University College London, London WC1E 6BT, UK; 36 Instituto de Alta Investigación, Tarapacá University, Arica 1000000, Chile; frothhammer@academicos.uta.cl; 37 Department of Biostatistics for Precision Oncology, Institut de Cancérologie Strasbourg Europe, 67200 Strasbourg, France

**Keywords:** gallbladder cancer, gallstone disease, genetic admixture, indigenous South American Mapuche ancestry, ancestry-informative markers, causal inference, instrumental variables, Mendelian randomization

## Abstract

**Simple Summary:**

An association between individual levels of indigenous Chilean Mapuche ancestry and the risk of gallbladder cancer has been reported in observational studies, and the current program for gallbladder prevention in Chile takes the number of Mapuche surnames into account for prophylactic cholecystectomy. However, the association between Mapuche ancestry and gallbladder cancer could be due to known and unknown confounders (e.g., overweight and obesity) and non-random measurement errors (e.g., socio-economic level and access to health care). To investigate whether Mapuche ancestry and gallbladder cancer risk are statistically correlated or causally related, we used ancestry-informative markers for instrumental variable analysis. We aim to provide robust evidence on the potential of accounting for ethnic differences (in this study, Mapuche ancestry) for disease interception (in this study, gallbladder cancer prevention through prophylactic cholecystectomy). The methodology used and the results obtained may also guide future admixture mapping studies.

**Abstract:**

A strong association between the proportion of indigenous South American Mapuche ancestry and the risk of gallbladder cancer (GBC) has been reported in observational studies. Chileans show the highest incidence of GBC worldwide, and the Mapuche are the largest indigenous people in Chile. We set out to assess the confounding-free effect of the individual proportion of Mapuche ancestry on GBC risk and to investigate the mediating effects of gallstone disease and body mass index (BMI) on this association. Genetic markers of Mapuche ancestry were selected based on the informativeness for assignment measure, and then used as instrumental variables in two-sample Mendelian randomization analyses and complementary sensitivity analyses. Results suggested a putatively causal effect of Mapuche ancestry on GBC risk (inverse variance-weighted (IVW) risk increase of 0.8% per 1% increase in Mapuche ancestry proportion, 95% CI 0.4% to 1.2%, *p* = 6.7 × 10^−5^) and also on gallstone disease (3.6% IVW risk increase, 95% CI 3.1% to 4.0%), pointing to a mediating effect of gallstones on the association between Mapuche ancestry and GBC. In contrast, the proportion of Mapuche ancestry showed a negative effect on BMI (IVW estimate −0.006 kg/m^2^, 95% CI −0.009 to −0.003). The results presented here may have significant implications for GBC prevention and are important for future admixture mapping studies. Given that the association between the individual proportion of Mapuche ancestry and GBC risk previously noted in observational studies appears to be free of confounding, primary and secondary prevention strategies that consider genetic ancestry could be particularly efficient.

## 1. Introduction

Every year, around 116,000 people are diagnosed with gallbladder cancer (GBC), and 85,000 die due to this aggressive disease worldwide [1]. The malignancy of the biliary tract primarily affects women in low- and middle-income countries, and relatively little effort has been invested in research on GBC [2,3].

The development of GBC is probably driven by a combination of environmental exposures and genetic predisposition [4,5]. Symptoms are often absent or unspecific until the disease has progressed to a non-curative stage, leaving patients with few treatment options [3,4,6]. As GBC is mostly diagnosed at an advanced stage, the 5-year survival rate is low. Studies report rates between 5% and 30% depending on the country of origin of the study population [3,4,7,8,9]. Gallbladder removal (cholecystectomy) is a valuable tool for primary and secondary prevention of GBC, but little progress has been made in individualized risk prediction and early diagnosis [6,10].

The incidence of GBC shows wide geographical and ethnic variation [2]. High-income regions such as Western Europe, the United States, and Australia have 1–2 cases per 100,000 person-years. In contrast, the largest indigenous people in Chile—the Mapuche—show the highest incidence of GBC in the world, with more than 20 cases per 100,000 person-years [11]. Observational studies have found a strong association between the individual proportion of indigenous American Mapuche ancestry and GBC risk: each 1% increase in the proportion of Mapuche ancestry translates into a 3.7% increase in GBC mortality [12]. However, the observed association may arise from other established GBC risk factors, especially gallstones and elevated body mass index (BMI) [3,4,6]. The Mapuche have a high prevalence of gallstone disease. A relative GBC risk of 4.9 has been observed in individuals with a history of gallstones, and recent studies have found evidence of a causal effect of gallstones on GBC risk for genetically admixed Chileans (odds ratio [OR] = 1.97) [2,3,13]. Indigenous American ancestry has also been associated with an increased BMI. The World Cancer Research Fund considers that higher body fatness marked by BMI probably causes GBC, and relative GBC risks of 1.59 for women and 1.09 for men per five-point increase in BMI have been reported [4,6,11,14].

In addition to any still unknown risk factors for GBC in high-incidence regions of South America, other potential confounders of the relationship between the individual proportion of Mapuche ancestry and GBC risk include socio-economic status and access to prophylactic cholecystectomy, multiparity in women, chronic inflammation, and lifestyle choices such as smoking and alcohol consumption [3,6,15,16]. The impossibility of accounting for unknown risk factors in standard statistical inference and the potential existence of non-random measurement errors in several confounders (e.g., accurate information on income and socio-economic level is difficult to obtain) justify the use of instrumental variables to assess the relationship between the proportion of Mapuche ancestry and GBC risk without confounding.

In response to the high GBC mortality rates and lack of treatment options, but based on weak scientific evidence, the Chilean government is implementing a GBC prevention program that provides financial support to gallstone patients for prophylactic cholecystectomy [6]. Prioritizing prophylactic cholecystectomy for those at high risk of GBC, taking into account unconfounded associations rather than observational correlations potentially attributable to confounding, would optimize the efficiency of current GBC prevention measures.

**Figure 1:**
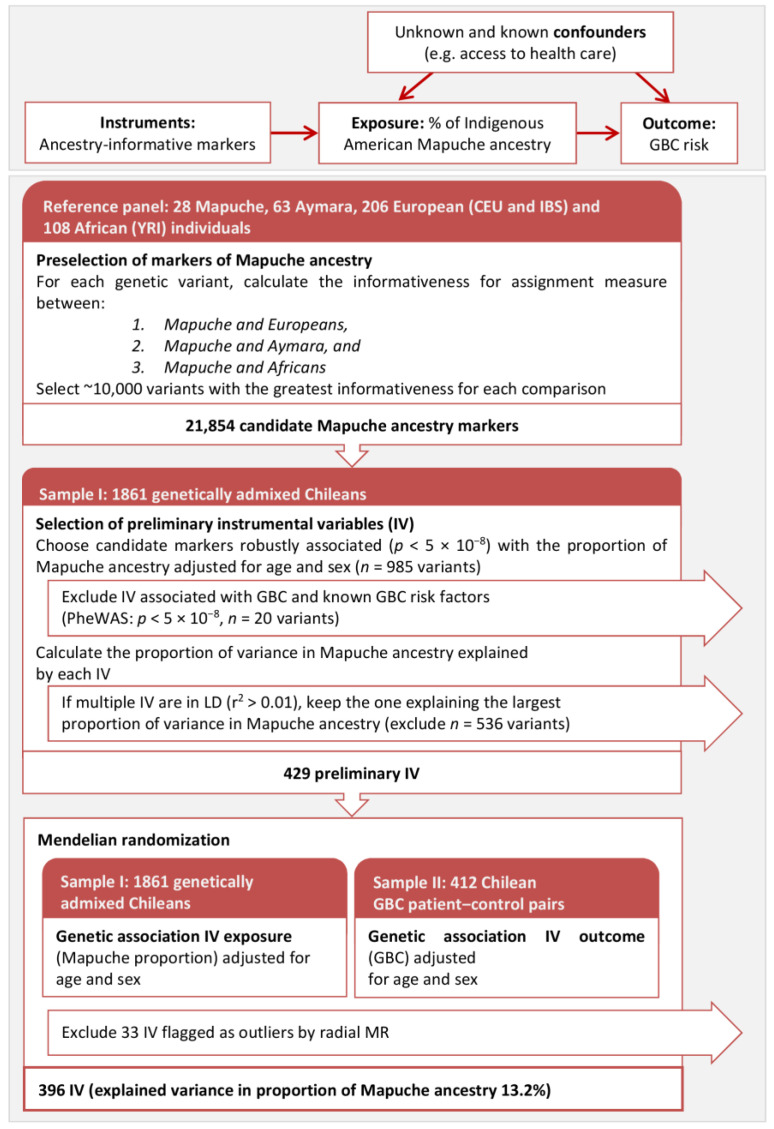
(**Upper panel**): The usual directed acyclic graph of Mendelian randomization (MR) adapted to the present study. (**Lower panel**): Flowchart describing the main analyses, from the selection of markers of Mapuche ancestry in a reference panel composed of Mapuche, Aymara, European, and African individuals to the two-sample MR based on 396 selected instrumental variables after exclusion of outlying instruments based on radial MR.

The upper part of Figure 1 shows the usual directed acyclic graph of Mendelian randomization (MR) adapted to the present study [17]. Increasing individual proportions of Mapuche ancestry (investigated exposure) have been associated with increasing GBC risk (investigated outcome), but this association could be due, at least in part, to unknown confounders and non-random measurement error in known confounders (e.g., socio-economic status or health care access).

To assess the confounding-free impact of Mapuche ancestry on GBC risk, we relied on the informativeness for assignment measure [18] to preselect markers of Mapuche ancestry in a panel of indigenous American, European, and African individuals. We then selected markers that were valid as instrumental variables: markers strongly associated with the individual proportion of Mapuche ancestry (relevance assumption), not associated with potential confounders (independence assumption), and not associated with GBC risk (exclusion restriction assumption; see Materials and Methods section for additional details). It should be noted that the individual proportion of indigenous Chilean ancestry is fixed at birth, but the alleles of the markers used as instrumental variables are randomly assigned during meiosis, mimicking a randomized trial.

## 2. Results

The flowchart in the lower part of Figure 1 describes the analyses performed with some intermediate results. The preselection of markers of Mapuche ancestry in a reference panel composed of indigenous American Mapuche (*n* = 28), Aymara (*n* = 63), European (CEU and IBS, *n* = 206), and African (YRI, *n* = 108) individuals relying on the informativeness for assignment measure yielded 21,854 candidate markers. Of these, 985 genetic variants were robustly associated (*p* < 5 × 10^−8^) with the individual proportion of Mapuche ancestry, which was previously estimated in a dataset with genome-wide genotype data from 1861 genetically admixed Chileans. Exclusion of markers associated with GBC or established GBC risk factors (PheWAS *p* < 5 × 10^−8^) complemented with LD pruning (r^2^ < 0.01) resulted in 429 instrumental variables being preliminarily retained for the subsequent MR analyses. Radial MR, based on summary statistics of the association between the instrumental variables and the proportion of Mapuche ancestry (sample I: 1861 admixed Chileans) and that between the instrumental variables and GBC status (sample II: 412 Chilean GBC patient-control pairs), detected 33 outlying instruments. The subsequent MR analysis of the association Mapuche ancestry → GBC was therefore based on 396 instrumental variables, which explained 13.2% of the variance in the proportion of Mapuche ancestry (F-statistic = 284.01).

**Figure 2:**
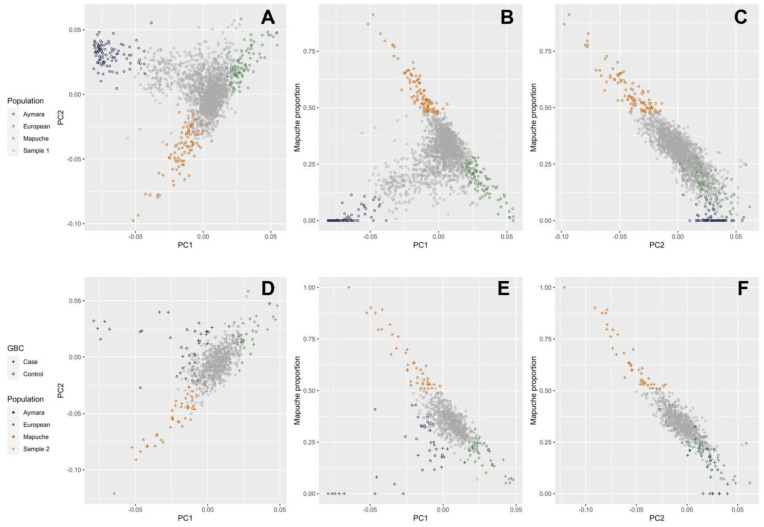
Scatter plots of the first and second genetic principal components (PC) and the individual proportion of Mapuche ancestry. Individuals with proportions of Mapuche, Aymara, and European ancestry above the 95th percentile are shown in orange, blue, and green, respectively. Panels (**A**–**C**) refer to sample I and panels (**D**–**F**) refer to sample II used in the main MR analysis, respectively. In sample II, GBC patients are represented by crosses and population-based controls by circles.

Figure 2 depicts the genetic principal components and the estimated proportions of Mapuche, Aymara, and European ancestry in the two samples used for the main MR analysis. Individuals with a proportion of Mapuche ancestry above the 95th percentile in the sample are represented in orange; blue and green highlight individuals with the largest proportions of Aymara and European ancestry, respectively. The first principal component in admixed Chileans explained a genetic variance of 1.2% and distinguished between European and indigenous Chilean ancestry. The second principal component explained a genetic variance of 0.2% and separated the two main types of indigenous American ancestry in Chile: Mapuche and Aymara (Figure 2A,D). A strong correlation between the second principal component and the proportion of Mapuche ancestry was found in the two samples used for MR analysis (Figure 2B,C,E,F). In the three plots depicting sample II (Figure 2D–F), crosses represent GBC patients and circles represent population-based controls. The concentration of GBC patients among individuals with the highest proportion of indigenous Chilean ancestry, especially Mapuche ancestry, was striking. Supplementary Figure S1 shows the distribution of individual estimated proportions of Mapuche, Aymara, European, and African ancestry.

**Figure 3:**
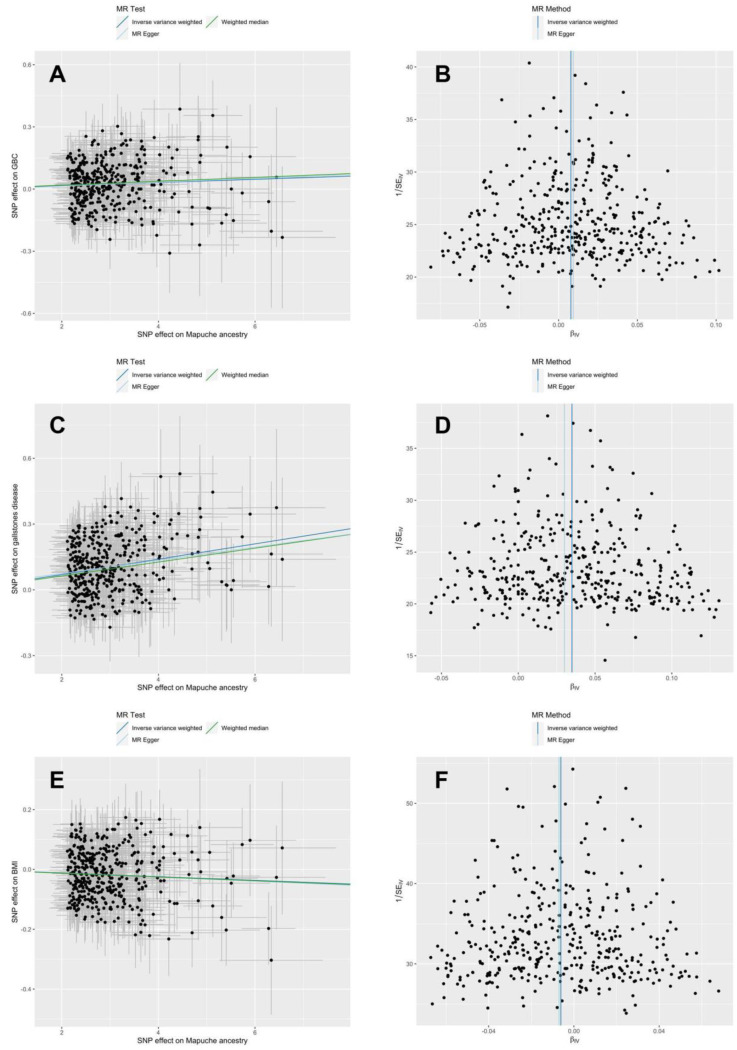
Scatter and funnel plots from MR analyses after exclusion of outlying instruments according to radial MR on the association between Mapuche ancestry and GBC risk (panels **A**,**B**), gallstone disease (panels **C**,**D**), and BMI (panels **E**,**F**).

Instrumental variable analysis of the association Mapuche ancestry → GBC risk revealed no heterogeneity among instruments as a proxy for pleiotropy (inverse variance-weighted (IVW) Cochran’s Q statistic *p* = 0.99) and no horizontal pleiotropy (MR-Egger intercept *p* = 0.87; Table 1). Neither outliers nor weak instrument biases were apparent in the scatter and funnel plots (Figure 3A,B). We found evidence of a putatively causal effect of Mapuche ancestry on GBC risk (IVW OR = 1.008, which translates into a GBC risk increase of 0.8% for every 1% increase in the proportion of Mapuche ancestry, 95% confidence interval [CI] 0.4% to 1.2%, *p* = 6.7 × 10^−5^; Table 1). MR-Egger (OR = 1.009, 95% CI 0.991 to 1.028, *p* = 0.33) and weighted median estimates (OR = 1.009, 95% CI 1.004 to 1.015, *p* = 1.0 × 10^−3^) were consistent with IVW estimates (Supplementary Table S1). As described above, the second principal component of genetic variability in admixed Chileans reflects the individual proportion of Mapuche ancestry, and, as expected, the causal effect vanished after including the second principal component in the calculation of summary statistics (*p* = 0.62; Table 1). In contrast, consideration of the first and third to tenth principal components translated into stronger estimates of the causal effect (1.4% risk increase, 95% CI 0.8% to 1.9%, *p* = 6.1 × 10^−7^). Steiger filtering and more stringent thresholds for LD pruning (r^2^ < 10^−3^) and PheWAS (*p* < 5 × 10^−6^) resulted in overlapping 95% CIs of the estimated effect sizes (Supplementary Table S1).

Further sensitivity analysis considered two-step MR and multivariable MR (MVMR) to investigate the potential mediating effects of gallstone disease and BMI on the link between Mapuche ancestry and GBC. For a two-step MR mediation analysis, we first assessed the unconfounded effect of Mapuche ancestry on gallstone disease. Neither heterogeneity among instrumental variables (Q statistic *p* = 0.95) nor horizontal pleiotropy (MR-Egger intercept *p* = 0.65; Table 1) was noted. MR results suggested a putatively causal effect of Mapuche ancestry on the risk of gallstone disease (IVW risk increase of 3.6% for every 1% increase in Mapuche proportion, *p* = 1.9 × 10^−59^; Table 1; MR-Egger OR = 1.031, 95% CI 1.010 to 1.052, *p* = 3.2 × 10^−3^; weighted median OR = 1.032, 95% CI 1.025 to 1.039, *p* = 1.2 × 10^−21^ (Supplementary Table S1). These results are visually represented in Figure 3C,D.

Since the proportion of Mapuche ancestry showed a confounding-free effect on gallstone disease in the present study and a causal effect of gallstones on GBC risk has already been demonstrated for Chileans, the two-step MR analysis suggests that gallstones mediate the association between Mapuche ancestry and GBC risk. To validate this hypothesis, we also performed MVMR. The instrumental variables used for MVMR were weak (Mapuche ancestry F-statistic = 3.5; gallstones F-statistic = 1.4), but horizontal pleiotropy was not found (Q statistic of 425 on 431 degrees of freedom, *p* = 0.57). In agreement with the results of two-step MR, MVMR revealed a causal effect of gallstones on GBC risk (OR = 1.263, *p* = 2.1 × 10^−9^) but no concurrent causal effect of Mapuche ancestry on GBC risk (OR = 1.0002, *p* = 0.95), suggesting complete mediation of the association between Mapuche ancestry and GBC risk by gallstones.

Neither heterogeneity among instrumental variables (Q statistic *p* = 0.95) nor horizontal pleiotropy (MR-Egger intercept *p* = 0.87; Table 1) was detected in the MR analysis of the association Mapuche ancestry → BMI. We found evidence of a negative, unconfounded effect of Mapuche ancestry on BMI (IVW β = −0.006 kg/m^2^ per 1% increase in Mapuche proportion, 95% CI −0.009 to −0.003, *p* = 5.0 × 10^−5^; Table 1). The corresponding scatter and funnel plots are shown in Figure 3E,F. MR-Egger (β = −0.007 kg/m^2^ per 1% Mapuche ancestry proportion, 95% CI −0.021 to 0.006, *p* = 0.30) and weighted median estimates (β = −0.006 kg/m^2^ per 1% Mapuche ancestry proportion, 95% CI −0.011 to −0.002, *p* = 7.0 × 10^−3^; Supplementary Table S1) showed good agreement with the negative effect estimated by IVW.

## 3. Discussion

By utilizing ancestry-informative markers as instrumental variables for the individual proportion of Mapuche ancestry in admixed Chileans, we investigated the confounding-free effect of Mapuche ancestry on GBC risk as the primary outcome and examined the mediating effects of gallstones and BMI on this association. According to MR results, the proportion of Mapuche ancestry showed an unconfounded effect on GBC development, which was mediated by gallstones. The practical implication of these results is that intensified surveillance and prophylactic gallbladder removal in gallstone carriers with a high percentage of Mapuche ancestry may improve the efficiency of current GBC prevention programs. Given the relationship between increasing proportions of Mapuche ancestry and decreasing BMI, and the putatively causal effect of BMI on GBC risk suggested by MR, it seems rather unlikely that BMI positively mediates between Mapuche ancestry and GBC risk.

Gallstones are highly prevalent in Chileans and one of the most important risk factors for GBC. Observational studies report a relative GBC risk of 9.2–10.1 for individuals with gallstones larger than 3 cm [3,19]. We therefore examined the possible mediating effect of gallstone disease on the association between Mapuche ancestry and GBC risk. The evidence for an unconfounded effect of Mapuche ancestry on gallstone disease was quite robust. The estimated causal OR varied from 1.031 (MR-Egger) to 1.036 (IVW) per 1% Mapuche ancestry proportion. One possible reason for this association would be genetic selection in Indigenous Americans towards efficient energy storage in periods of nutritional and caloric insufficiency, which now, under conditions of energy abundance, translates into increased susceptibility to gallstone formation [20]. The unconfounded relationship between the individual proportion of Mapuche ancestry and gallstones, together with the previously reported causal effect of gallstones on GBC risk in Chileans, supports a mediating effect of gallstones based on two-step MR [13]. The MVMR results, based on a relatively small sample size and weak instrumental variables, showed good agreement with two-step MR and pointed to a fully mediating effect of gallstones between Mapuche ancestry and GBC risk. However, large prospective Chilean datasets with complete information on GBC, gallstones, and individual genotype data are urgently needed to conduct formal mediation analyses and accurately quantify the magnitude of direct and indirect effects, which are highly relevant for more precise GBC prevention.

The International Agency for Research on Cancer, the American Institute for Cancer Research, and the World Cancer Research Fund all consider obesity to be a likely cause of GBC, and high BMI is another important GBC risk factor, with a direct effect on GBC risk reported for Chileans and an indirect effect mediated by gallstones for Europeans [13,14,21]. However, studies of the association between indigenous American ancestry and BMI have yielded contradictory results. BMI decreased by 0.13 m/kg^2^ for each 10% increase in the proportion of indigenous American ancestry in Mexican-American women [22], and the association was also negative in a Hispanic-Mexican study [23]. In contrast, BMI increased by 0.56 m/kg^2^ per 10% increase in the proportion of indigenous ancestry in Indigenous Americans [24]. Indigenous American ancestry was positively correlated with obesity in Mexico and Peru, whereas no association was found in Brazil, Chile, or Colombia, and higher Indigenous American ancestry was associated with overweight and obesity, but only among foreign-born Latina women [25]. In the present study, we found clear evidence of a negative confounding-free effect of Mapuche ancestry on BMI, which probably rules out a positive mediating effect of BMI on the association between Mapuche ancestry and GBC risk. However, further in-depth analyses in larger studies are needed to quantify the magnitude of the unconfounded effect of the individual proportion of Mapuche ancestry on BMI.

The relatively small number of GBC patients investigated was a limitation of this study, especially considering the large sample sizes normally required for MR. However, observational studies have reported a strong association between the individual proportion of Mapuche ancestry and GBC mortality risk (3.7% increased GBC mortality risk per 1% increase in Mapuche ancestry), and the variance in the proportion of Mapuche ancestry explained by the instrumental variables was relatively high. It should be noted that, in order to be able to rule out potential confounding by instrumental variable analysis, we did not directly investigate the association between individual proportions of Mapuche ancestry (100% total variance) and GBC risk but rather between genetic variants strongly associated with the individual proportion of Mapuche ancestry (13.2% explained variance) and GBC risk. In addition to low statistical power, another common limitation of MR studies is pleiotropy. First-order inverse variance weights keep the type I error rate under the causal null, and we calculated Cochran’s Q statistic using first-order weights to detect heterogeneity, which often reflects pleiotropy. We also visually inspected scatter and funnel plots, performed MR-Egger regression to detect potential bias attributable to horizontal pleiotropy, used radial MR to detect outlying instruments, and excluded instrumental variables associated with GBC and GBC risk factors not investigated in our study.

Previous studies have demonstrated the importance of subdividing indigenous American ancestry into its main subcomponents; in the case of admixed Chileans, the two major indigenous American subcomponents are Mapuche and Aymara ancestry [12]. While combined indigenous Chilean ancestry showed no association with GBC mortality and the proportion of Aymara ancestry showed a negative association with GBC mortality, each 1% increase in the Mapuche proportion translated into a 3.7% increase in the risk of death due to GBC (95% CI 3.1 to 4.3%). To assess the contribution of health system access to the identified association between Mapuche ancestry and GBC risk, hospitalization rates due to gallbladder removal (cholecystectomy) were previously included as an additional explanatory variable in standard regression models. However, the standard approach to statistical adjustment is limited by (1) potentially large, non-random measurement errors in the adjustment variables (e.g., information on socio-economic level and access to health care) and (2) the impossibility of adjusting for as yet unknown GBC risk factors, which may be specific to regions with high GBC incidence. Without the need to know, accurately measure, and statistically adjust for all GBC risk factors, the instrumental variable-based OR estimated in this study points to an unconfounded association between Mapuche ancestry and GBC risk, suggesting that GBC prevention strategies that consider the individual proportion of Mapuche ancestry could be particularly efficient.

Beyond their practical relevance for GBC prevention, the results presented here may also be useful for designing future studies. On the one hand, the evidence of a putatively causal effect of Mapuche ancestry on GBC risk underpins the potential of admixture mapping to identify novel GBC susceptibility variants, possibly in combination with subsequent association testing (note that the genome-wide significance level is much higher for admixture mapping than for association mapping) [26]. For example, assuming that the average proportion of Mapuche ancestry in Chileans is about 40%, approximately 1100 GBC patients are needed to detect a Mapuche haplotype with a risk ratio of 1.8, consistent with the estimated causal OR of 1.008 per 1% proportion of Mapuche ancestry in the whole Chilean genome [27]. On the other hand, the 3.7% increased GBC mortality risk per 1% Mapuche ancestry proportion previously reported in observational studies probably overestimates the contribution of Mapuche ancestry to GBC risk and would lead to underpowered admixture mapping studies. From an implementation perspective, recruiting study participants from the southern regions of Chile would increase the average proportion of Mapuche ancestry and thus the statistical power of the study.

## 4. Materials and Methods

**Patient and public involvement:** A representative of the Chilean Foundation of Gastrointestinal Cancer Patients (www.gist.cl, accessed on 3 August 2023) reviewed the informed consent forms and is a permanent member of the External Advisory Board of the European-Latin American Consortium towards Eradication of Preventable Gallbladder Cancer—EULAT Eradicate GBC (www.SaludVesiculaBiliar.org, accessed on 3 August 2023), which meets annually to discuss the project objectives, the progress of the project, and the relevance of the project results to patients. The EULAT Eradicate GBC dissemination videos are also available in Mapudungun, the language of the Mapuche people (https://youtu.be/HsPeid1Rmus, accessed on 3 August 2023). Our study did not include Mapuche individuals directly, but genetically admixed Chileans who consented to the use of their genetic data for the present investigation. The manuscript was reviewed by an expert in Mapuche culture from the Universidad de la Frontera in Temuco, Chile. This study spans genetics, epidemiology, public health, and anthropology. Interdisciplinary collaborations are essential to advance our understanding of complex diseases and improve current prevention, early detection, and treatment [28,29]. We recently organized a symposium on Native American Ancestry in Genetic Research Projects at the joint meeting of the Chilean Genetics Society and the Chilean Society of Evolution and are organizing the summer school “Ancestry meets Molecular Health” (https://www.klinikum.uni-heidelberg.de/medizinische-biometrie/forschung/arbeitsgruppen/statistical-genetics/research-training, accessed on 3 August 2023) to discuss how to involve communities and individuals in the design and implementation of genetic research projects and improve the dissemination of study results.

**Study participants:** The Chilean genome is a mixture of chromosomal segments from two major indigenous American peoples, the Aymara in the north and the Mapuche in the south of the country; Europeans; and, to a lesser extent, Africans [4,30,31]. The reference panel for preselection of ancestry-informative markers and estimation of individual ancestry proportions in genetically admixed Chileans therefore included 63 Aymara [32] and 28 Mapuche [32,33], as well as 206 Europeans (99 Utah residents with Northern and Western European ancestry [CEU] and 107 Iberians from Spain [IBS]), and 108 African Yorubans from Ibadan, Nigeria (YRI) from the 1000 Genome Project [34].

Sample I for two-sample MR analyses of the associations Mapuche ancestry → GBC, Mapuche ancestry → gallstone disease and Mapuche ancestry → BMI consisted of 1861 Chileans from the Consortium for the Analysis of the Diversity and Evolution of Latin America (CANDELA) [30]. Sample II for MR analysis of the association between Mapuche ancestry and GBC risk was composed of 412 Chilean patients diagnosed with GBC and 412 population-based controls matched by age and sex with the GBC patients. Sample II for MR analysis of the association between Mapuche ancestry and gallstone disease was composed of 351 Chilean gallstone patients and 351 controls matched by age and sex. Among GBC patients, 77% were diagnosed after surgical removal of the gallbladder (cholecystectomy), and gallstones were found in about 86% of the GBC patients investigated. Gallstone patients were patients who underwent cholecystectomy due to symptomatic gallstones. Population-based controls were selected from the Chilean subgroup of CANDELA and from Chilean studies on COPD and Chagas disease, with GBC and gallstone incidences representative of the general Chilean population [10]. Information on family history of GBC was available for patients with GBC and gallstones, but not for population-based controls. Sample I and sample II for MR analyses on Mapuche ancestry → GBC risk and Mapuche ancestry → gallstone disease partially overlapped: 84 of the 412 controls and 91 of the 351 controls in the respective sample II also belonged to sample I. Sample II for MR analysis of the association between Mapuche ancestry and BMI was based on 12,216 individuals from the Hispanic Community Health Study/Study of Latinos (HCHS/SOL, dbGaP accession number phs000810.v1.p1).

**Preselection of markers of Mapuche ancestry:** To preselect genetic variants robustly associated with our exposure of interest, the individual proportion of Mapuche ancestry, we first chose markers of Mapuche ancestry based on the informativeness for assignment measure *I_n_* [18]. For each genetic variant with *j* = 1, …, *N* possible alleles in *i* = 1, …, *K* subpopulations, we calculated:

(1) $$I_{n}\left(Q,J\right)=\sum_{j=1}^{N}\left(-p_{j}\log\left(p_{j}\right)+\sum_{i=1}^{K}\frac{p_{ij}}{K}\log\left(p_{ij}\right)\right),$$

where *p_j_* denotes the average frequency of allele *j* in all *K* subpopulations, *p_ij_* the average frequency of allele *j* in subpopulation *i*, *Q* the (random) assignment of an individual to a subpopulation, and *J* the (random) genotype of one of the two alleles of an individual. *I_n_* conditioning on *Q* and *J* is a general approach to quantifying the amount of information that multi-allelic markers provide about individual ancestry composition. We used the reference panels described above to estimate *I_n_* for each of the 43,625 genetic variants available for all individuals in this study, considering two subpopulations at a time (Mapuche-European, Mapuche-Aymara, and Mapuche-African). We selected the 10,000 genetic variants with the highest *I_n_* for each comparison and retained the genetic variants that were present in at least one comparison for the subsequent analyses.

**Selection of instrumental variables:** MR relies on the random assortment of genetic variants during meiosis, which mimics a randomized trial and thus circumvents potential confounding. Individual proportions of genetic ancestry are fixed at birth, but the alleles of the ancestry-informative markers used as instrumental variables in this study were randomly assigned at meiosis. To ensure that the preselected ancestry-informative markers were valid instrumental variables fulfilling the first MR assumption (relevance), we used the software ADMIXTURE (version 1.3) for supervised estimation of the individual proportions of Mapuche ancestry in sample I, relying on the reference panel of Aymara, Mapuche, European, and African individuals [35], and retained as instrumental variables (IV) the genetic variants that showed a robust association (*p* < 5 × 10^−8^) with the estimated proportion of Mapuche ancestry, adjusted for age and sex. The second MR assumption (independence) was assessed by a phenome-wide association study (PheWAS), excluding genetic variants associated (*p* < 5 × 10^−8^) with GBC risk or potential confounders (menopause, educational level, diabetes, body circumference, smoking, alcohol consumption, or gallstones) in MR-Base [36]. We then used the summary statistics of the association between the genetic variants and the estimated proportion of Mapuche ancestry (*β*) and the minor allele frequency (MAF) to calculate the variance in Mapuche ancestry proportion explained by each variant [37] using the equation:

(2) $$explained\;variance=\beta^{2}\times 2\times MAF\times \left(1-MAF\right).$$

We determined the pairwise linkage disequilibrium between genetic variants using the R package ‘genetics’ [38] in sample I, composed of genetically admixed Chileans, and excluded variants in linkage disequilibrium (LD) (r^2^ > 0.01) with other variants that explained a larger variance. The explained variance, I_n_s, and subpopulation allele frequencies for the 50 genetic variants that explained the greatest variance in Mapuche ancestry proportion are shown in Supplementary Table S2.

In an attempt to fulfill the third MR assumption (exclusion restriction), radial MR was applied separately for each investigated outcome to detect and subsequently exclude outlying and influential genetic variants in combination with further techniques described in the next section.

**Two-sample MR, sensitivity, and mediation analyses (two-step and multivariable MR):** For the previously selected instrumental variables, we calculated summary statistics of genetic association with the individual proportion of Mapuche ancestry in sample I and summary statistics of genetic association with GBC, gallstones, and BMI in the respective sample II. We fitted linear (proportion of Mapuche ancestry and BMI) or logistic (GBC and gallstone disease) regression models, assuming an additive genotype model and considering age and sex as covariates. We then used the summary statistics to examine the association between Mapuche ancestry and GBC risk using two-sample MR as the primary analysis. To guarantee instrumental validity, we also (1) visually inspected the funnel and scatter plots of summary statistics to detect weak instrument bias; (2) calculated Cochran’s Q statistic using first-order inverse variance weights to detect heterogeneity, which indicates a possible violation of the instrumental variable or modeling assumptions, of which pleiotropy is a likely major cause; and (3) used the *p*-value for a non-zero MR-Egger intercept to assess horizontal pleiotropy. Our primary objective was to identify statistical causal effects, which require weaker MR assumptions than estimation of their magnitude, and we tested causality based on random-effect inverse variance-weighted (IVW) *p*-values. As a secondary objective, we estimated the causal effect sizes and assessed their robustness by comparing IVW, weighted median, and MR-Egger regression parameter estimates (ORs for GBC and gallstone disease and beta values for BMI per 1% increase in the proportion of Mapuche ancestry).

Population stratification is particularly relevant in MR studies of genetically admixed individuals. To deal with potential stratification, association statistics are typically adjusted for the main principal components of genetic variability. However, the individual proportion of Mapuche ancestry was the exposure of interest in this study of Chileans, who show continuous gradients of ancestry. To check the sensitivity of MR results to population stratification, we estimated the genetic principal components in samples I and II using the eigenstrat function [39], examined the correlation between the estimated principal components and the proportion of Mapuche ancestry, adjusted the association statistics for (1) all of the first ten principal components or (2) the first ten principal components with the exception of the principal component correlated with the proportion of Mapuche ancestry, and repeated the MR analyses. Additional sensitivity analyses included Steiger filtering to ensure that the instrumental variables explained a larger variance in the exposure than in the investigated outcome [40] and applying stricter thresholds for LD pruning (r^2^ < 10^−3^) and PheWAS (*p* < 5 × 10^−6^).

Since a causal effect of gallstones and BMI on GBC risk has recently been reported [13], we investigated the mediating effects of gallstone disease and BMI as a surrogate marker for obesity as potential mechanisms explaining the relationship between Mapuche ancestry and GBC risk. The unavailability of large prospective Chilean datasets with complete information on GBC, gallstones, BMI, and individual genotypes precluded the implementation of formal mediation analyses, and we decided first to apply two-step MR to assess mediation [41]. In the first MR step, instrumental variables for the proportion of Mapuche ancestry were used to assess the causal effect of Mapuche ancestry on the potential mediator (gallstone disease or BMI) by MR as described above. The second MR step was based on published findings, also based on genetically admixed Chileans, on the causal effect of the mediators on GBC risk. Evidence of association in the two MR steps (Mapuche ancestry → gallstone disease and gallstone disease → GBC) would imply some degree of mediation between Mapuche ancestry and GBC risk on the part of the intermediate trait (gallstones). We also performed MVMR to simultaneously assess the causal effects of Mapuche ancestry and gallstones on GBC risk. MVMR allows the estimation of mediation effects when considering potentially correlated risk factors using genetic instruments within the MR framework. We used the R package ‘MVMR’, following the workflow suggested by the software developers [42]. We calculated F statistics to monitor instrument strength, Q statistics to assess instrument validity, including horizontal pleiotropy, and finally estimated the direct effects of the considered exposures (Mapuche ancestry and gallstone disease) simultaneously on the outcome of interest (GBC risk). The instrumental variables used included the previously selected ancestry-informative markers along with five genetic variants (one palindromic variant was removed) robustly associated with gallstone disease in Chileans [43]. Sample I and sample II used to assess the genetic associations with Mapuche ancestry proportion and gallstone disease did not overlap. They consisted of 1703 Chileans (1861 minus those used as controls in sample II) and 351 gallstone patients and matched controls, respectively. The sample used to assess the direct effect between instrumental variables and gallstone disease on GBC risk was the same as the sample II utilized for the MR analysis of Mapuche ancestry → GBC risk (412 Chilean GBC cases and 412 matched controls).

MR analyses were conducted using the R version of MR-Base, which provides convenient tools for the harmonization of the association statistics, including standardization of the effect alleles and removal of problematic palindromic genetic variants [36]. The R package ‘RadialMR’ was used for radial MR [44]. For general data processing, we used Plink version 1.9 [45] and the R software environment for statistical computing and graphics (version 3.6.2).

## 5. Conclusions

In summary, we assessed the confounding-free relationship between the individual proportion of indigenous Chilean Mapuche ancestry and GBC risk using ancestry-informative markers as instrumental variables. To our knowledge, this is the first MR study that considers the proportion of genetic ancestry as the exposure of interest. It is important to keep in mind that the study was based on genetically admixed Chileans who showed continuous gradients of Mapuche ancestry. Since evidence of an unconfounded effect of ancestry proportion on disease development is generally more relevant than an observational correlation, the present findings provide more refined information on the potential of accounting for ethnic differences (in this case Mapuche ancestry) in disease prevention (in this case GBC). Individual ancestry proportions are not a modifiable exposure, but individuals with a high proportion of Mapuche ancestry could be prioritized for gallstone screening and prophylactic cholecystectomy—after extensive complementary research into potential side effects and cost-effectiveness. Future admixture mapping studies could also benefit from the methodology applied in the present investigation to test the confounding-free effect of genetic ancestry on disease outcomes. From a more applied point of view, instrumental variable results suggested a putatively causal effect of Mapuche ancestry proportion on GBC risk, most likely mediated by gallstones, with direct implications for the development of more efficient GBC prevention strategies.

## Figures and Tables

**Table 1:** MR results on the causal relationship between the proportion of Mapuche ancestry as exposure, and GBC, gallstone disease, and BMI as outcomes of interest. Cochran’s Q statistic *p*-values higher than 0.05 are suggestive of no instrument heterogeneity as a proxy for pleiotropy, and MR-Egger intercept *p*-values higher than 0.05 are consistent with no horizontal pleiotropy. Results from sensitivity analyses on the association between Mapuche ancestry → GBC risk after including the first to the tenth principal component, both with and without the second principal component, which reflects the individual proportion of Mapuche ancestry in admixed Chileans, are also shown.

		Inverse Variance Weighted					MR-Egger
Outcome	IV	β/OR	95% CI		β *p*-Value	Q *p*-Value	Intercept *p*-Value
GBC	396	1.008	1.004	1.012	**6.7 × 10^−5^**	0.99	0.87
Including PC1-PC10	396	1.007	0.979	1.037	0.62	0.99	0.17
Including PC1 and PC3-PC10	396	1.014	1.008	1.019	**6.1 × 10^−7^**	0.99	0.36
Gallstone disease	387	1.036	1.031	1.040	**1.9 × 10^−59^**	0.95	0.65
BMI	390	−0.006	−0.009	−0.003	**5.0 × 10^−5^**	0.95	0.87

IV: Number of instrumental variables; β/OR: estimated causal effect size for BMI/estimated causal odds ratio for GBC and gallstone disease per 1% increase in the proportion of Mapuche ancestry; CI: confidence interval; Q: Cochran’s Q statistic; PC: principal components estimated based on individual genotypes. Bold represents probability values smaller than 0.05.

## Data Availability

The source code to reproduce all the results described is provided as supplementary material, and the necessary input files with the summary association statistics are available at www.biometrie.uni-heidelberg.de/StatisticalGenetics/Software_and_Data (accessed on 3 August 2023).

## Supplementary Materials

The following supporting information can be downloaded at: https://www.mdpi.com/article/10.3390/cancers15164033/s1, Table S1: Results from sensitivity MR analysis on the relationship between the proportion of indigenous American Mapuche ancestry as investigated exposure and GBC, gallstone disease and BMI as outcomes of interest. Table S2: Overview of the 50 genetic variants used as instrumental variables for Mendelian randomization analysis that explain the greatest variance in Mapuche ancestry proportion. Figure S1: Bar plot of the results from admixture analyses for individuals in Sample I and Sample II, as well as in sample II divided into GBC patients and healthy controls.

## References

[1] The Global Cancer Observatory Global Cancer Observatory: Cancer Today—Gallbladder Cancer Fact Sheet. https://gco.iarc.fr/today/data/factsheets/cancers/12-Gallbladder-fact-sheet.pdf.

[2] Mehrotra R., Tulsyan S., Hussain S., Mittal B., Singh Saluja S., Singh S., Tanwar P., Khan A., Javle M., Hassan M.M. (2018). Genetic Landscape of Gallbladder Cancer: Global Overview. Mutat. Res.-Rev. Mutat. Res..

[3] Stinton L.M., Shaffer E.A. (2012). Epidemiology of Gallbladder Disease: Cholelithiasis and Cancer. Gut Liver.

[4] Hundal R., Shaffer E.A. (2014). Gallbladder Cancer: Epidemiology and Outcome. Clin. Epidemiol..

[5] Pérez-Moreno P., Riquelme I., García P., Brebi P., Roa J.C. (2022). Environmental and Lifestyle Risk Factors in the Carcinogenesis of Gallbladder Cancer. J. Pers. Med..

[6] Roa I., de Aretxabala X. (2015). Gallbladder Cancer in Chile: What Have We Learned?. Curr. Opin. Gastroenterol..

[7] Kanthan R., Senger J.-L., Ahmed S., Kanthan S.C. (2015). Gallbladder Cancer in the 21st Century. J. Oncol..

[8] Bertran E., Heise K., Andia M.E., Ferreccio C. (2010). Gallbladder Cancer: Incidence and Survival in a High-Risk Area of Chile. Int. J. Cancer.

[9] Zhu X., Zhang X., Hu X., Ren H., Wu S., Wu J., Wu G., Si X., Wang B. (2020). Survival Analysis of Patients with Primary Gallbladder Cancer from 2010 to 2015: A Retrospective Study Based on SEER Data. Medicine.

[10] Blandino A., Scherer D., Rounge T.B., Umu S.U., Boekstegers F., Barahona Ponce C., Marcelain K., Gárate-Calderón V., Waldenberger M., Morales E. (2022). Identification of Circulating LncRNAs Associated with Gallbladder Cancer Risk by Tissue-Based Preselection, Cis-EQTL Validation, and Analysis of Association with Genotype-Based Expression. Cancers.

[11] Campbell P.T., Newton C.C., Kitahara C.M., Patel A.V., Hartge P., Koshiol J., McGlynn K.A., Adami H.-O., Berrington De Gonzalez A., Beane Freeman L.E. (2017). Body Size Indicators and Risk of Gallbladder Cancer: Pooled Analysis of Individual-Level Data from 19 Prospective Cohort Studies. Cancer Epidemiol. Biomark. Prev..

[12] Lorenzo Bermejo J., Boekstegers F., González Silos R., Marcelain K., Baez Benavides P., Barahona Ponce C., Müller B., Ferreccio C., Koshiol J., Fischer C. (2017). Subtypes of Native American Ancestry and Leading Causes of Death: Mapuche Ancestry-Specific Associations with Gallbladder Cancer Risk in Chile. PLoS Genet..

[13] Barahona Ponce C., Scherer D., Brinster R., Boekstegers F., Marcelain K., Gárate-Calderón V., Müller B., de Toro G., Retamales J., Barajas O. (2021). Gallstones, Body Mass Index, C-Reactive Protein, and Gallbladder Cancer: Mendelian Randomization Analysis of Chilean and European Genotype Data. Hepatology.

[14] World Cancer Research Fund/American Institute for Cancer Research Diet, Nutrition, Physical Activity and Cancer: A Global Perspective. Continuous Update Project Expert Report 2018. http://dietandcancerreport.org.

[15] Lugo A., Peveri G., Gallus S. (2020). Should We Consider Gallbladder Cancer a New Smoking-Related Cancer? A Comprehensive Meta-Analysis Focused on Dose–Response Relationships. Int. J. Cancer.

[16] Wistuba I.I., Gazdar A.F. (2004). Gallbladder Cancer: Lessons from a Rare Tumour. Nat. Rev. Cancer.

[17] Sanderson E., Glymour M.M., Holmes M.V., Kang H., Morrison J., Munafò M.R., Palmer T., Schooling C.M., Wallace C., Zhao Q. (2022). Mendelian Randomization. Nat. Rev. Methods Prim..

[18] Rosenberg N.A., Li L.M., Ward R., Pritchard J.K. (2003). Informativeness of Genetic Markers for Inference of Ancestry. Am. J. Hum. Genet..

[19] Miquel J.F., Covarrubias C., Villaroel L., Mingrone G., Greco A.V., Puglielli L., Carvallo P., Marshall G., Del Pino G., Nervi F. (1998). Genetic Epidemiology of Cholesterol Cholelithiasis among Chilean Hispanics, Amerindians, and Maoris. Gastroenterology.

[20] Carey M.C., Paigen B. (2002). Epidemiology of the American Indians’ Burden and Its Likely Genetic Origins. Hepatology.

[21] Lauby-Secretan B., Scoccianti C., Loomis D., Grosse Y., Bianchini F., Straif K. (2016). Body Fatness and Cancer—Viewpoint of the IARC Working Group. N. Engl. J. Med..

[22] Hu H., Huff C.D., Yamamura Y., Wu X., Strom S.S. (2015). The Relationship between Native American Ancestry, Body Mass Index and Diabetes Risk among Mexican-Americans. PLoS ONE.

[23] Tang H., Jorgenson E., Gadde M., Kardia S.L.R., Rao D.C., Zhu X., Schork N.J., Hanis C.L., Risch N. (2006). Racial Admixture and Its Impact on BMI and Blood Pressure in African and Mexican Americans. Hum. Genet..

[24] Norden-Krichmar T.M., Gizer I.R., Libiger O., Wilhelmsen K.C., Ehlers C.L., Schork N.J. (2014). Correlation Analysis of Genetic Admixture and Social Identification with Body Mass Index in a Native American Community. Hum. Biol..

[25] Ziv E., John E.M., Choudhry S., Kho J., Lorizio W., Perez-Stable E.J., Burchard E.G. (2006). Genetic Ancestry and Risk Factors for Breast Cancer among Latinas in the San Francisco Bay Area. Cancer Epidemiol. Biomark. Prev..

[26] Horimoto A.R.V.R., Xue D., Thornton T.A., Blue E.E. (2021). Admixture Mapping Reveals the Association between Native American Ancestry at 3q13.11 and Reduced Risk of Alzheimer’s Disease in Caribbean Hispanics. Alzheimer’s Res. Ther..

[27] McKeigue P.M. (2005). Prospects for Admixture Mapping of Complex Traits. Am. J. Hum. Genet..

[28] Arango-Isaza E., Capodiferro M.R., Aninao M.J., Babiker H., Aeschbacher S., Achilli A., Posth C., Campbell R., Martínez F.I., Heggarty P. (2023). The Genetic History of the Southern Andes from Present-Day Mapuche Ancestry. Curr. Biol..

[29] Moreno-Mayar J.V. (2023). Human Genetics: Rich Genomic History of Two Isolated Indigenous Peoples of South America. Curr. Biol..

[30] Ruiz-Linares A., Adhikari K., Acuña-Alonzo V., Quinto-Sanchez M., Jaramillo C., Arias W., Fuentes M., Pizarro M., Everardo P., de Avila F. (2014). Admixture in Latin America: Geographic Structure, Phenotypic Diversity and Self-Perception of Ancestry Based on 7,342 Individuals. PLoS Genet..

[31] Eyheramendy S., Martinez F.I., Manevy F., Vial C., Repetto G.M. (2015). Genetic Structure Characterization of Chileans Reflects Historical Immigration Patterns. Nat. Commun..

[32] Reich D., Patterson N., Campbell D., Tandon A., Mazieres S., Ray N., Parra M.V., Rojas W., Duque C., Mesa N. (2012). Reconstructing Native American Population History. Nature.

[33] Lindo J., Haas R., Hofman C., Apata M., Moraga M., Verdugo R.A., Watson J.T., Llave C.V., Witonsky D., Beall C. (2018). The Genetic Prehistory of the Andean Highlands 7000 Years BP Though European Contact. Sci. Adv..

[34] (2015). The 1000 Genomes Project Consortium. A Global Reference for Human Genetic Variation. Nature.

[35] Alexander D.H., Novembre J., Lange K. (2009). Fast Model-Based Estimation of Ancestry in Unrelated Individuals. Genome Res..

[36] Hemani G., Zheng J., Elsworth B., Wade K.H., Haberland V., Baird D., Laurin C., Burgess S., Bowden J., Langdon R. (2018). The MR-Base Platform Supports Systematic Causal Inference across the Human Phenome. eLife.

[37] Brion M.J.A., Shakhbazov K., Visscher P.M. (2013). Calculating Statistical Power in Mendelian Randomization Studies. Int. J. Epidemiol..

[38] Warnes G.R. (2003). The Genetics Package—Utilities for Handling Genetic Data. R News.

[39] Patterson N., Price A.L., Reich D. (2006). Population Structure and Eigenanalysis. PLoS Genet..

[40] Hemani G., Tilling K., Davey Smith G. (2017). Orienting the Causal Relationship between Imprecisely Measured Traits Using GWAS Summary Data. PLoS Genet..

[41] Zheng J., Baird D., Borges M.-C., Bowden J., Hemani G., Haycock P., Evans D.M., Davey Smith G. (2017). Recent Developments in Mendelian Randomization Studies. Curr. Epidemiol. Rep..

[42] Sanderson E., Spiller W., Bowden J. (2021). Testing and Correcting for Weak and Pleiotropic Instruments in Two-Sample Multivariable Mendelian Randomization. Stat. Med..

[43] Joshi A.D., Andersson C., Buch S., Stender S., Noordam R., Weng L.C., Weeke P.E., Auer P.L., Boehm B., Chen C. (2016). Four Susceptibility Loci for Gallstone Disease Identified in a Meta-Analysis of Genome-Wide Association Studies. Gastroenterology.

[44] Bowden J., Spiller W., Del Greco F.M., Sheehan N., Thompson J., Minelli C., Smith G.D. (2018). Improving the Visualization, Interpretation and Analysis of Two-Sample Summary Data Mendelian Randomization via the Radial Plot and Radial Regression. Int. J. Epidemiol..

[45] Purcell S., Neale B., Todd-Brown K., Thomas L., Ferreira M.A.R., Bender D., Maller J., Sklar P., de Bakker P.I.W., Daly M.J. (2007). PLINK: A Tool Set for Whole-Genome Association and Population-Based Linkage Analyses. Am. J. Hum. Genet..

