# Supplementary material for: Gallbladder Cancer Risk and Indigenous South American Mapuche Ancestry: Instrumental Variable Analysis Using Ancestry-Informative Markers
Source: Cancers (Basel). 2023 Aug 9;15(16):4033. doi: 10.3390/cancers15164033 (PMC10452561; doi:10.3390/cancers15164033)
Supplement: Supplementary file 1 [file cancers-15-04033-s001.zip › cancers-2471665-supplementary.pdf]

Supplementary Table S1. Results from sensitivity MR analysis on the relationship between the proportion of indigenous American Mapuche ancestry as investigated exposure, and GBC, gallstone disease and BMI as outcomes of interest. Cochran's Q statistic *p*-values higher than 0.05 are suggestive of no instrument heterogeneity as a proxy for pleiotropy, and MR-Egger intercept *p*-values higher than 0.05 are consistent with no horizontal pleiotropy. Results including Steiger filtering and obtained using alternative thresholds for LD pruning and PheWAS are also shown.

Outcome	Method	IV	Exp. Var.	β/OR	95% CI		β *p*-Value	Q *p*-Value	Intercept *p*-Value
GBC	IVW	396	0.132	1.008	1.004	1.012	**6.7×10^{-5}**	0.99	
	Egger	396		1.009	0.991	1.028	0.33	0.99	0.87
	WM	396		1.009	1.004	1.015	**1.0×10^{-3}**		
Steiger filtering	IVW	266	0.095	1.004	1.000	1.009	0.06	0.99	
LD $r^2 < 10^{-3}$	IVW	65	0.027	1.009	1.000	1.017	0.05	0.90	
PheWAS $p < 5 \times 10^{-6}$	IVW	387	0.128	1.008	1.004	1.012	**2.7×10^{-5}**	0.99	
Gallstones disease	IVW	387	0.128	1.036	1.031	1.040	**1.9×10^{-59}**	0.95	
	Egger	387		1.031	1.010	1.052	**3.2×10^{-3}**	0.95	0.65
	WM	387		1.032	1.025	1.039	**1.2×10^{-21}**		
BMI	IVW	390	0.128	−0.006	−0.009	−0.003	**5.0×10^{-5}**	0.95	
	Egger	390		−0.007	−0.021	0.006	0.30	0.95	0.87
	WM	390		−0.006	−0.011	−0.002	**7.0×10^{-3}**		

IV: Number of instrumental variables; Exp. Var.: variance explained by the instrumental variables; β/OR: estimated causal effect size for BMI/estimated causal odds ratio for GBC and gallstone disease per 1% increase in proportion of Mapuche ancestry; CI: confidence interval; Q: Cochran's Q statistic; Steiger filtering: additional Steiger filtering before linkage disequilibrium (LD) pruning of IV; IVW: Inverse variance weighted; Egger: MR-Egger; WM: Weighted median estimates; LD $r^2 < 10^{-3}$: more stringent thresholds for LD pruning; PheWAS $p < 5 \times 10^{-3}$: more stringent thresholds for phenome wide association study (PheWAS). Bold type shows probability values smaller than 0.05.

Supplementary Table S2. Overview of the 50 genetic variants used as instrumental variables for Mendelian randomization analysis that explained the greatest variance in Mapuche ancestry proportion. The table shows the explained variance, I_n estimates for two subpopulations at a time (Mapuche–European, Mapuche–Aymara and Mapuche–African) and the minor allele frequencies (MAF) in the reference panels.

Genetic Variant	Variance Explained [%]	I_n Mapuche-Aymara	I_n Mapuche-European	I_n Mapuche-African	MAF (Mapuche)	MAF (Aymara)	MAF (European)	MAF (African)
rs3107547	0.0891	0.0037	0.0660	0.0277	0.071	0.032	0.595	0.301
rs9790655	0.0879	0.0002	0.0012	0.0362	0.143	0.159	0.095	0.449
rs3796349	0.0816	0.0262	0.0076	0.0359	0.214	0.056	0.391	0.028
rs573712	0.0802	0.0113	0.0019	0.0017	0.125	0.254	0.197	0.176
rs10875652	0.0748	-0.0001	0.0024	0.0025	0.143	0.143	0.226	0.088
rs9295957	0.0734	0.0227	0.0150	0.0093	0.161	0.365	0.405	0.306
rs12139288	0.0705	0.0005	0.0746	0.0823	0.375	0.341	0.891	0.838
rs10434645	0.0680	0.0002	0.0010	0.0361	0.339	0.318	0.277	0.088
rs4785798	0.0660	0.0006	0.0001	0.0118	0.089	0.111	0.078	0.232
rs7942935	0.0654	0.0081	0.0123	0.0042	0.518	0.381	0.745	0.630
rs11090362	0.0652	0.0008	0.0547	0.1821	0.018	0.032	0.444	0.699
rs1566816	0.0623	0.0131	0.0205	0.0007	0.036	0	0.262	0.056
rs2099131	0.0609	0.0195	0.0126	0.0066	0.446	0.246	0.223	0.588
rs1891292	0.0600	0.0003	0.0088	0.0447	0.411	0.437	0.614	0.764
rs7007884	0.0597	0.0001	0.0549	0.1015	0.143	0.135	0.636	0.681
rs2759280	0.0587	0.0380	0.0014	0.0040	0.339	0.103	0.265	0.241
rs7497935	0.0580	0.0017	-0.0001	0.0083	0.107	0.071	0.104	0.032
rs2961766	0.0576	0.0020	0.0039	0.0432	0	0.008	0.034	0.213
rs520226	0.0561	0.0044	0.0558	0.0390	0.554	0.452	0.114	0.227
rs1352230	0.0558	0.0025	0.0035	0.0158	0.125	0.079	0.056	0.023
rs8058531	0.0552	0.0061	0.0261	0.0849	0	0.024	0.209	0.380
rs2885856	0.0541	0.0102	0.0005	0.0001	0.125	0.246	0.095	0.130
rs6671606	0.0530	0.0068	0.0083	0.2158	0.054	0.127	0.187	0.810
rs6956460	0.0529	0.0005	0.0287	0.0031	0.107	0.087	0.442	0.176
rs4987774	0.0528	0.0174	0.0145	0.0041	0.661	0.460	0.401	0.551
rs11590987	0.0527	0.0103	0.0455	0.0243	0	0.040	0.337	0.125
rs12665552	0.0525	0.0124	0.0117	0.2625	0	0.048	0.100	0.806
rs2757069	0.0523	0.0021	0.0132	0.0025	0.482	0.413	0.248	0.569
rs7425956	0.0521	0.0001	0.0027	0.0092	0.482	0.468	0.371	0.319
rs12878905	0.0515	0.0059	0.0062	0.0572	0.554	0.437	0.384	0.171
rs194072	0.0515	0.0213	0.0094	0.0229	0.107	0.286	0.019	0.329
rs10238377	0.0513	0.0015	0.0010	0.0033	0.315	0.254	0.376	0.407
rs17124108	0.0513	0.0434	0.1059	0.2997	0.071	0.325	0.740	0.926
rs2051090	0.0508	0.0009	0.0249	0.1691	0.018	0.008	0.257	0.671
rs6802463	0.0499	0.0013	0.0064	0.0058	0.143	0.183	0.289	0.250
rs2791384	0.0494	0.0017	0.0180	0.1679	0.036	0.063	0.243	0.699
rs12562944	0.0494	0.0023	0.0432	0.0041	0.143	0.198	0.578	0.232
rs3788372	0.0485	0.0023	0.0001	0.0738	0.214	0.278	0.194	0
rs12044217	0.0482	0.0120	0.0014	0.1111	0.268	0.429	0.342	0.819
rs2896165	0.0477	0.0005	0.0001	0.0027	0.036	0.024	0.044	0.079
rs11014059	0.0477	0.0088	0.0011	0.0251	0.214	0.111	0.158	0.046
rs7694527	0.0472	0.0145	0.0121	0.1776	0	0.056	0.102	0.648
rs16944600	0.0471	0.0650	0.1132	0.0975	0.643	0.262	0.053	0.144
rs2432545	0.0465	0.0070	0.0109	0.0178	0.268	0.389	0.490	0.495
rs10202112	0.0462	0.0046	0.0062	0.0506	0	0.016	0.049	0.236

rs899747	0.0461	0.0145	0.0158	0.1331	0.232	0.405	0.498	0.833
rs4904019	0.0461	0.0020	0.0439	0.0381	0	0.008	0.328	0.190
rs1294884	0.0459	0.0002	0.0178	0.0850	0.231	0.191	0.512	0.736
rs1564996	0.0456	0.0026	0.0041	0.0343	0.196	0.262	0.320	0.023
rs11128254	0.0456	0.0061	0.0079	0.0392	0	0.024	0.068	0.194

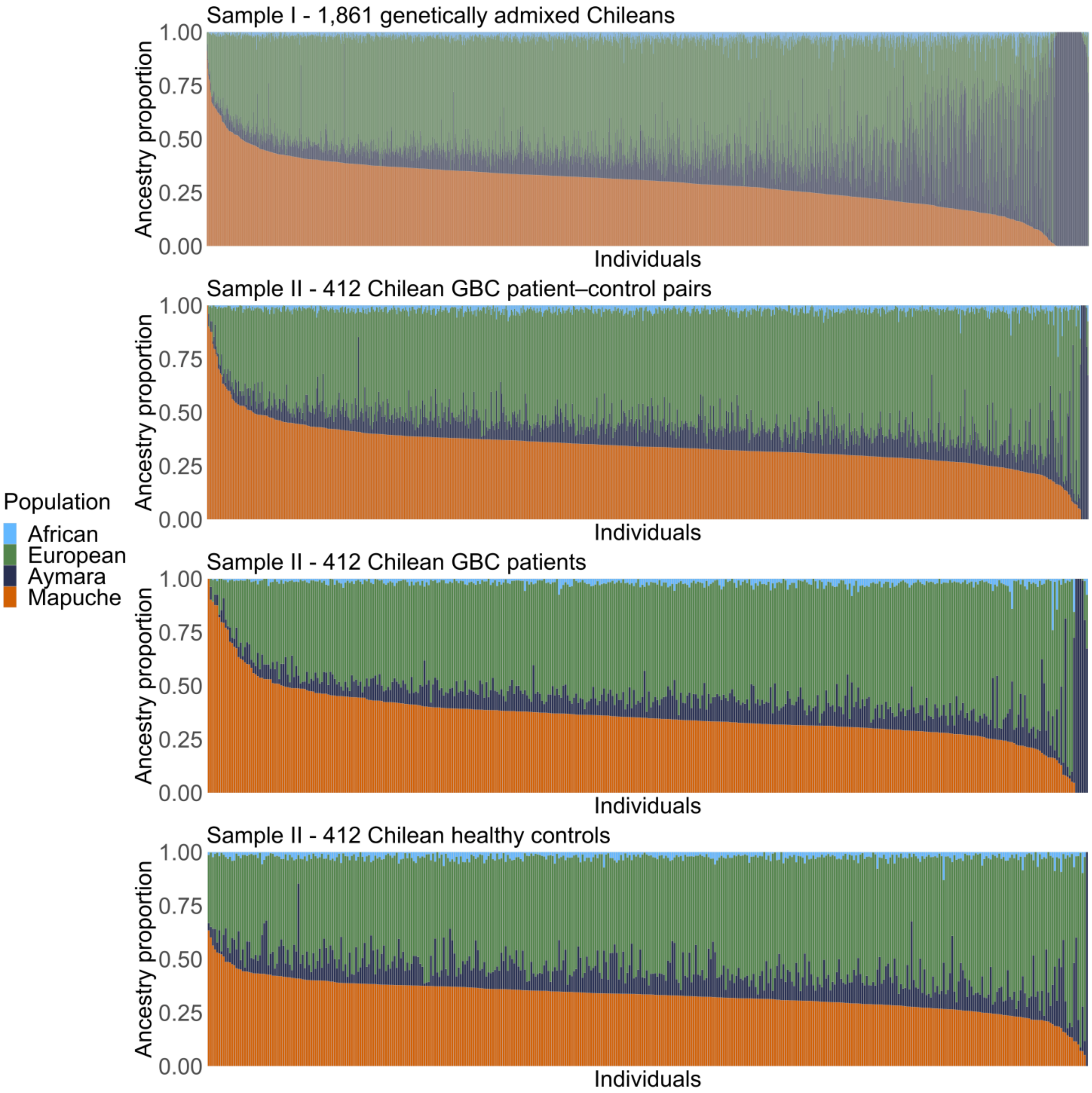

Supplementary Figure S1. Bar plot of the results from admixture analyses for individuals in Sample I and Sample II, as well as in sample II divided into GBC patients and healthy controls. The proportions of Mapuche, Aymara, European and African ancestry are shown in orange, dark blue, green and light blue, respectively.
